# Modelling, Bayesian inference, and model assessment for nosocomial pathogens using whole‐genome‐sequence data

**DOI:** 10.1002/sim.8510

**Published:** 2020-03-06

**Authors:** Rosanna Cassidy, Theodore Kypraios, Philip D. O'Neill

**Affiliations:** ^1^ School of Mathematical Sciences University of Nottingham Nottingham UK

**Keywords:** antimicrobial resistance, Bayesian methods, MCMC, MRSA, whole‐genome sequences

## Abstract

Whole‐genome sequencing of pathogens in outbreaks of infectious disease provides the potential to reconstruct transmission pathways and enhance the information contained in conventional epidemiological data. In recent years, there have been numerous new methods and models developed to exploit such high‐resolution genetic data. However, corresponding methods for model assessment have been largely overlooked. In this article, we develop both new modelling methods and new model assessment methods, specifically by building on the work of Worby et al. Although the methods are generic in nature, we focus specifically on nosocomial pathogens and analyze a dataset collected during an outbreak of MRSA in a hospital setting.

## 1 INTRODUCTION

Recent years have seen intense research activity directed towards methods for analyzing data on outbreaks of communicable diseases where the data contain high‐resolution genetic information, such as whole‐genome sequences. Particular attention has been given to methods for reconstructing transmission trees.^1^, ^2^, ^3^, ^4^, ^5^, ^6^, ^7^, ^8^, ^9^ Broadly speaking, such methods fall into two categories, namely those which require an initial reconstruction of a phylogenetic tree, which itself may be topologically dissimilar to the transmission tree itself,^10^ and those which do not. Among the latter are those in which statistical inference is carried out by defining a probability model conditional on the observed data, meaning that there is no underlying model that fully describes how the data were generated. For example, a probability model for possible transmission trees can be defined conditional upon observed symptom appearance times, but with no explicit model for the times themselves.^6^, ^11^ Conversely, both Lau et al^12^ and Worby et al^1^ provide such data‐generating models that incorporate both the transmission dynamics of the epidemic and the genetic component. The Lau et al model assumes an underlying model for the within‐host evolution of the pathogen, whereas the Worby et al model uses a phenomenological model for the observed genetic distances in the data. One advantage of the latter approach is that it avoids detailed assumptions about microevolution processes, which are often not well‐understood.

An attractive aspect of using data‐generating models is that they can be used to assess the model fit by quantifying how plausible the observed data are under the proposed model. However, to our knowledge, there have been no attempts to date to develop model assessment techniques for transmission tree reconstruction methods which involve some kind of statistical model. The only partial exception is in Worby et al^1^ in which a Bayesian posterior predictive approach is used to assess model fit, but the focus is on the epidemiological aspects of the observed data rather than the genetic part. One objective of the current article is to develop a model assessment method for high‐resolution genetic data.

Roughly speaking, the models described in Worby et al^1^ are defined by taking a standard individual‐based stochastic epidemic model, such as a susceptible‐infective‐removed model, and then generating a random distance between each pair of infective individuals. Such a distance represents a genetic difference between the pathogen in the two individuals, and its distribution depends on the relationship between the individuals in the transmission tree. A typical genetic distance model assumes that distances between pathogens will be positively correlated with some measure of the individuals' separation in the transmission tree. However, the Worby et al models draw genetic distances in a completely independent manner, which is somewhat unrealistic. For example, in an infection chain of individuals in which *A* infects *B* infects *C*, one might reasonably expect that the genetic distance between the pathogens in *A* and *C* should not be independent of the *AB* and *BC* distances. A second objective of this article is to provide new genetic distance models, which overcome this problem by incorporating a natural dependence structure.

Our methods will be illustrated via application to a patient‐level dataset taken from an outbreak of methicillin‐resistant *Staphylococcus aureus* (MRSA) in a hospital in Thailand. The data include both epidemiological information such as the admission and discharge times of patients, and the dates and results of screening tests, and also genetic information in the form of whole‐genome‐sequence data taken from isolates. The latter include examples of multiple isolates taken from the same patient. Our analysis will provide estimates of both transmission rates and likely transmission routes of the pathogen.

The article is structured as follows. The transmission model and associated genetic distance models are introduced in Section 2, and inference methods are described in Section 3. Model assessment methods are described in Section 4, along with an associated simulation study. The MRSA dataset and subsequent analysis can be found in Section 5 and we finish with conclusions and discussion in Section 6.

## 2 STOCHASTIC TRANSMISSION MODELS WITH GENETIC COMPONENTS

We now describe a general stochastic model that describes both the transmission of a pathogen within a single hospital ward and the way in which observed genetic distances between isolates arise. The model contains parameters which will be estimated using data that consist of admission and discharge times of individual patients, and the dates and results of diagnostic tests to detect the pathogen, the latter including genetic data. Since our focus is not on the timing of admissions, discharges or diagnostic tests, the model will assume such events to be determined by the data. Some of the underlying assumptions of the model are discussed in more detail in Section 6.

### 2.1 Transmission model

The model is discrete‐time with days as time‐units. We assume a study period starting on day *T*_S_ and ending on day *T*_E_. The ward is assumed to consist of a fixed number of beds, each of which may be empty or be assigned to one patient. As mentioned above, the times at which patients enter and leave the ward are assumed to be known from data, and thus can be regarded as deterministic events within the model.

At any time, each patient present on the ward is either *susceptible*, meaning that they are free from the pathogen in question, or else *colonized*, meaning that they carry the pathogen at a detectable level. Note that colonization status only refers to the presence of the pathogen and does not indicate whether or not the patient has any symptoms or illness as a result of colonization. We assume that once a patient is colonized, they remain so for the remainder of their time on the ward. Each patient who enters the ward is, independently of all other patients, assumed to be already colonized with probability *p*, and otherwise susceptible. Patients who enter the ward as colonized are said to be *colonized on admission*.

Patients who are colonized are able to colonize susceptible patients who are on the ward at the same time. In reality such transmission of the pathogen is likely to be indirect, for instance via healthcare workers who attend the patients on the ward. We assume that each susceptible patient on day *t* avoids colonization on that day with probability exp(−βC(t)), where *C*(*t*) denotes the number of colonized patients on the ward on day *t*, and otherwise is colonized. If colonization occurs, then (i) the susceptible patient is regarded as being colonized on day *t*+1 and able to colonize other patients and (ii) the patient responsible for the transmission event, who we refer to as the *source* of the event, is selected uniformly at random from the *C*(*t*) colonized patients on the ward. Patients who become colonized via transmission events on the ward are said to be *colonized on the ward*.

Our assumptions regarding transmission correspond to homogenous mixing insofar as every colonized patient is equally likely to be able to colonize any susceptible patient. Note also that exp(−β) is the probability that a given susceptible patient avoids colonization from a given colonized patient during a single day.

### 2.2 Diagnostic tests and genetic distances

Whilst on the ward, patients may have diagnostic tests to identify the pathogen. Following Worby et al^1^ we assume that the tests have perfect specificity, so that a susceptible patient never tests positive, and sensitivity *z*, meaning that a colonized patient has probability *z* of testing positive. The assumption of perfect specificity can easily be relaxed if required. Test outcomes are assumed to be mutually independent given the underlying colonization states. Some of the isolates obtained via tests may be sequenced. Note that a single patient may have multiple sequenced isolates.

In order to construct a model that describes genetic distances between isolates, that is, between observed sequences, we instead define a more general model that describes distances between all sequences, whether they are observed or not. An implicit assumption is that each colonized patient has one associated sequence if they either have zero or one isolate, or *n* sequences if they have *n*≥2 isolates.

If a patient *A* has a sequence *i* as a result of an isolate obtained on day *t*, then draw a distance ψ_*i*,*j*_ to each sequence *j* generated on or before day *t*, where ψ_*i*,*j*_ is a realization of a nonnegative integer‐valued random variable Ψ_*i*,*j*_. Here, Ψ_*i*,*j*_ may depend on both the relative position of the patients associated with *i* and *j* in the chain of transmission between them, if any, and other genetic distances already generated. Specific examples of Ψ_*i*,*j*_ are given in Section 2.4. Note that *A* may have multiple sequences due to tests on different days, and for each one we generate associated distances to other sequences. Conversely, for a patient *B* who first enters colonized status on day *t* and never has an isolate taken, we suppose that they have an unobserved sequence *i* on day *t* and draw distances to all sequences *j* generated on day *t* or earlier in the same manner as for patient *A*.

Note that although we have described the generation of genetic distances as occurring through time as the outbreak unfolds according to the transmission model, it is also possible to generate the distances conditional upon the entire outbreak, since the transmission dynamics do not explicitly depend on the distances. Either way, the genetic distances have to be generated in time‐order if Ψ_*i*,*j*_ allows dependencies on existing genetic distances, which is the case for the models in this article.

### 2.3 Transmission forest and transmission distance

Recall that the model description includes sources, that is, the identities of patients responsible for transmission events. Thus the model also specifies the *transmission forest*, that is, a directed graph made up of disconnected components, each of which has a tree structure in which nodes correspond to colonized patients and an edge from one node to another corresponds to a transmission event. The root of each tree corresponds to a patient who is colonized on admission. We refer to a directed path starting at one node and terminating at another as a *transmission chain*.

For two sequences *i* and *j*, respectively, associated with patients *A* and *B* we define the *transmission distance k*=*k*(*i*,*j*)=*k*(*j*,*i*) to be the length of the transmission chain, if any, from *A* to *B* or vice versa in the transmission forest. Thus *k*=1 if *A* colonized *B* or vice versa, *k*=2 if *A* colonized *C* who colonized *B* or vice versa, and so on. We set *k*=∞ if there is no such transmission chain; note that this is automatically true if *A* and *B* are in different trees, but can also be true if *A* and *B* are in the same tree. For example, if *C* colonizes *A* and *B*, then there is no directed path from *A* to *B* or vice versa and so *k*=∞. We also define *k*=0 if *A* and *B* are the same patient, in order to account for patients who have multiple sequenced isolates.

Suppose now that *k*(*i*,*j*)>1 and that the transmission chain from *A* to *B* is *A*,*C*_1_,…,*C*_*m*_,*B* for some *m*≥1. For *k*=1,…,*m* denote by σ(*k*) the first (ie, earliest in time) sequence associated with patient *C*_*k*_, and define

$$D=D(i,j)=\psi_{i,\sigma (1)}+\overset{m-1}{\underset{k=1}{\sum }}\psi_{\sigma (k),\sigma (k+1)}+\psi_{\sigma (m),j},$$

where ψ_*k*,*l*_ denotes the genetic distance between *k* and *l*. Thus *D* is the sum of the genetic distances associated with direct colonization events along the chain, where we take one pair of sequences for each such event. We will use *D* to define Ψ_*i*,*j*_ in a way that incorporates dependencies on existing genetic differences.

### 2.4 Specific models for genetic distances

We now provide two basic models for the Ψ_*i*,*j*_ random variable used to generate genetic distances. Both models involve the Poisson distribution, which is natural in this context if one assumes that the genetic mutations leading to differences between sequences are rare events in some sense. However, other desired distributions can also be used, as illustrated in the MRSA application in Section 5. Both models also include an explicit dependence on existing genetic differences, unlike the models described in Worby et al^1^ in which all Ψ_*i*,*j*_ values are mutually independent.

#### 2.4.1 The Poisson error dependence model

The first new model, the Poisson error model, assumes that the genetic distance between sequences *i* and *j* follows a Poisson distribution with parameter θ_*G*_, θ_*I*_, or θ if the corresponding patients are, respectively, not connected by a transmission chain (*k*(*i*,*j*)=∞), the same patient (*k*(*i*,*j*)=0), or adjacent in a transmission chain (*k*(*i*,*j*)=1). It is also assumed that all these distances are mutually independent. Conversely if *k*(*i*,*j*)>1, the genetic distance is defined as *D*(*i*,*j*)+*ξW*, where *P*(ξ=1)=*P*(ξ=−1)=0.5, *W* is a Poisson random variable with parameter *k*(*i*,*j*)γ truncated at *D*(*i*,*j*), and ξ and *W* are independent. The truncation ensures that the genetic distance cannot be negative. The motivation for this part of the model is that Ψ_*ij*_ will equal *D*(*i*,*j*) on average, and have a variance that will increase with *k*(*i*,*j*). It follows that for *x*=0,1,…,

(1) $$P(\Psi_{i,j}=x)=\left\{\begin{array}{cc} (\theta_{G}^{x}/x!)\exp(-\theta_{G})\; & \text{if}\;k(i,j)=\infty ,\\ (\theta_{I}^{x}/x!)\exp(-\theta_{I})\; & \text{if}\;k(i,j)=0,\\ (\theta^{x}/x!)\exp(-\theta )\; & \text{if}\;k(i,j)=1,\\ \frac{{\left(k(i,j)\gamma \right)}^{\left|x-D(i,j)\right|}}{|x-D(i,j)|!C_{D}}{\left(\frac{1}{2}\right)}^{1_{\left\{x\neq D(i,j)\right\}}}1_{\left\{x\leq 2D(i,j)\right\}}\; & \text{if}\;k(i,j)>1, \end{array}\right.$$

where 1_*A*_ denotes the indicator function of the event *A*, and $C_{D}=\sum_{l=0}^{D(i,j)}{\left(k\gamma \right)}^{l}/l!$. Note that although Equation (1) only specifies the marginal distribution of each Ψ_*i*,*j*_, the joint distribution is simply the product of (i) the marginal distributions for *k*(*i*,*j*)=0,1 and ∞ and (ii) the marginal distributions for *k*(*i*,*j*)>1 conditional on (i). An explicit formula for the joint distribution is given in Section 3.2.

#### 2.4.2 The Poisson chain dependence model

Our second model has a similar structure to the first but now assumes that for sequences *i* and *j* where *k*(*i*,*j*)>1, the genetic distance is simply modelled as a Poisson random variable with mean *D*(*i*,*j*). Thus Ψ_*ij*_ will equal *D*(*i*,*j*) on average, and with a variance that will increase with *D*(*i*,*j*). For *x*=0,1,…, we define

(2) $$P(\Psi_{i,j}=x)=\left\{\begin{array}{cc} (\theta_{G}^{x}/x!)\exp(-\theta_{G})\; & \text{if}\;k(i,j)=\infty ,\\ (\theta_{I}^{x}/x!)\exp(-\theta_{I})\; & \text{if}\;k(i,j)=0,\\ (\theta^{x}/x!)\exp(-\theta )\; & \text{if}\;k(i,j)=1,\\ (D{\left(i,j\right)}^{x}/x!)\exp(-D(i,j))\; & \text{if}\;k(i,j)>1. \end{array}\right.$$

## 3 INFERENCE METHODS

We now describe methods for fitting our models to data. We use a Bayesian framework and employ data‐augmented Markov chain Monte Carlo (MCMC) methods.

### 3.1 Data

We assume that the available data contain three components, denoted ***y***, ***x***, and **ψ**. Component ***y*** is the set of dates of admission and discharge, plus the dates of any diagnostic tests, for every patient in the study. These dates are assumed to be known accurately and we make no attempt to model them. Component ***x*** is the set of results, that is, positive or negative, of all diagnostic tests. Component **ψ** is the set of sequenced isolates obtained during the study. For our purposes it is sufficient for this to be summarized as the set of all observed genetic distances $\psi =\left\{\psi_{i,j}:i<j\right\}$. Such distances are typically obtained by counting the number of single nucleotide polymorphisms (SNPs) between a pair of sequences.^1^, ^8^

It is possible for a single patient to be admitted to the ward several times during the study. For simplicity, we regard such readmissions as being different patients in the sense that we take no explicit account of a patient's previous history if they are readmitted. In other words, we will use the term *patient* to refer to *patient episode*. However, our methods can easily be extended to introduce dependencies between readmissions of the same patient, for instance by assuming that a patient previously colonized will still be colonized if readmitted within a given length of time.^13^ In practice, the benefit of such additional modelling depends on the proportion of admissions that are readmissions.

### 3.2 Bayesian inference and data augmentation

Both models defined in Section 2 have parameters $\rho =\left\{p,\beta ,z,\Theta \right\}$, where Θ denotes the parameters of the genetic distance model. In a Bayesian framework, the object of interest is the posterior density π(ρ|***x***,***y***,**ψ**)∝π(***x***,**ψ**|***y***,ρ)π(ρ), where π(***x***,**ψ**|***y***,ρ) is the likelihood and π(ρ) is the prior density of ρ, assumed to be independent of ***y*** a priori. However, the likelihood is analytically and computationally intractable in practice, because its evaluation involves summing over all possible colonization events and unobserved sequences, both of which are found in the underlying stochastic model. We therefore proceed by introducing additional parameters *T* and **ψ** ^*u*^, corresponding to unobserved colonization events and unobserved genetic sequences, in order to obtain a tractable augmented likelihood. Specifically we use the decomposition

(3) $$\pi (\rho ,T,\psi^{u}|x,y,\psi )\propto \pi (x,\psi ,\psi^{u}|\rho ,T,y)\pi (T|\rho ,y)\pi (\rho ).$$

Here, π(*T*|ρ,***y***) is the likelihood of colonization events, while π(***x***,**ψ**,**ψ** ^*u*^|ρ,*T*,***y***) is the likelihood of the test results and both observed and unobserved genetic differences conditioned on the colonization events.

Let 𝒫 denote the set of all patients in the study. For patient *k*, let $t_{k}^{a}$ and $t_{k}^{d}$ denote, respectively, the date of their admission and discharge from the ward. If *k* is ever colonized set $t_{k}^{c}$ as the date on which they first enter the colonized state, and otherwise set $t_{k}^{c}=\infty$. Note that $t_{k}^{c}$ is not observed, and neither is the actual number of colonized patients, since a colonized patient may avoid detection by never having a diagnostic test or by testing negative. For patient *k*, define *ϕ*_*k*_=1 if *k* is colonized on admission and *ϕ*_*k*_=0 otherwise, and let ${𝒫}^{c}=\left\{k\in 𝒫:\phi_{k}=0,t_{k}^{c}\neq \infty \right\}$ denote the set of patients who are colonized on the ward. For patient $k\in {𝒫}^{c}$, set *s*_*k*_=*l* if *k* is colonized by source patient *l*. Let $𝒞(t)=\left\{k\in 𝒫:t_{k}^{c}\leq t\leq t_{k}^{d}\right\}$ denote the set of patients in the colonized state on day *t*. Thus, the number of colonized patients on day *t* is given by C(t)=|𝒞(t)|.

Define $t^{c}=\left\{t_{k}^{c}:k\in 𝒫\right\}$, $\phi =\left\{\phi_{k}:k\in 𝒫\right\}$, $s=\left\{s_{k}:k\in {𝒫}^{c}\right\}$ and define **ψ** ^*u*^ as the set of genetic distances involving unobserved sequences. Finally, let $T=\left\{t^{c},\phi ,s\right\}$.

Under the assumption of perfect specificity of the diagnostic test, each positive test in the data ***x*** must be a true positive. Given *T*, we can also evaluate the number of false negative tests in ***x*** since we know the true colonization status of every patient at every time. Denote the numbers of true positive and false negative tests by TP and FN, respectively. Then the first term on the right‐hand side of Equation (3) is

(4) $$\pi (x,\psi ,\psi^{u}|\rho ,T,y)=z^{\text{TP}}{\left(1-z\right)}^{\text{FN}}P\left(\underset{(i,j)\in 𝒮}{⋂}\left\{\Psi_{i,j}=\psi_{i,j}\right\}\right),$$

where $𝒮=\left\{(i,j):i<j,\;\psi_{i,j}\in \psi \cup \psi^{u}\right\}$ is the set of all pairs of sequences.

The joint distribution of genetic distances can be evaluated as

$$\begin{array}{ll} P\left(\underset{(i,j)\in 𝒮}{⋂}\left\{\Psi_{i,j}=\psi_{i,j}\right\}\right)=\left(\underset{(i,j)\in {𝒮}_{1}}{\prod }P\left(\Psi_{i,j}=\psi_{i,j}\right)\right)\left(\underset{(i,j)\in {𝒮}_{2}}{\prod }P\left(\Psi_{i,j}=\psi_{i,j}|\left\{\Psi_{u,v}=\psi_{u,v}:(u,v)\in {𝒮}_{1}\right\}\right)\right), & \end{array}$$

where ${𝒮}_{1}=𝒮\cap \left\{(i,j):k(i,j)\in \left\{0,1,\infty \right\}\right\}$ and ${𝒮}_{2}=𝒮\cap \left\{(i,j):k(i,j)>1\right\}$, and where the terms in the products are given by (1) or (2) depending on the choice of model.

The likelihood of colonization events is given by

(5) $$\begin{array}{ll} \pi (T|\rho ,y) & =p^{\sum_{k}\phi_{k}}{\left(1-p\right)}^{\sum_{k}(1-\phi_{k})}\;\\ & \;\times \underset{k\in 𝒫}{\prod }\left[1_{\left\{t_{k}^{c}=t_{k}^{a}\right\}}+1_{\left\{t_{k}^{c}\neq t_{k}^{a}\right\}}\exp\left(-\overset{\min(t_{k}^{c}-1,t_{k}^{d})}{\underset{t=t_{k}^{a}}{\sum }}\beta C(t)\right)\right]\;\\ & \;\times \underset{l\in {𝒫}^{c}}{\prod }\left(\frac{1-\exp(-\beta C(t_{l}^{c}))}{C(t_{l}^{c})}\right)1_{\left\{s_{l}\in 𝒞(t)\right\}}.\; \end{array}$$

The three terms on the right hand side of Equation (5) give the probabilities of (i) the admission status of each patient, (ii) patients avoiding colonization, and (iii) patients being colonized by the source specified in ***s***. Note that the indicator function ensures that the source of a patient *l* who is colonized on the ward must themselves be colonized when *l* becomes colonized; otherwise, the likelihood will be zero.

### 3.3 Markov chain Monte Carlo methods

In order to explore the posterior density defined at Equation (3) , we use an MCMC algorithm to sample from it. Our setting is sufficiently complex to make the use of standard MCMC software packages infeasible in practice. The algorithm updates in turn the epidemiological parameters (*p*,*z*, and β), the genetic parameters Θ, and the latent (ie, unobserved) variables *T* and **ψ** ^*u*^. Our algorithm is related to that described in Worby et al,^1^ but includes some extensions and refinements as well as a number of additional steps to improve the mixing properties of the resulting Markov chain. Full details of the algorithm can be found in the supplementary material, but here we describe it in outline for the Poisson chain dependence model.

All assigned prior distributions are assumed to be mutually independent. We assume a priori that *p* and *z* follow Beta distributions, β and γ follow improper Uniform prior distributions on (0,∞), and θ,θ_*I*_,θ_*G*_ follow Gamma distributions. We use the notation ρ_−*p*_ to denote ρ with *p* removed, and so on.

Algorithm 1 **MCMC algorithm to sample from *π*(*ρ*,*T*,*ψ* ^*u*^|*x*,*y*,*ψ*)**

1

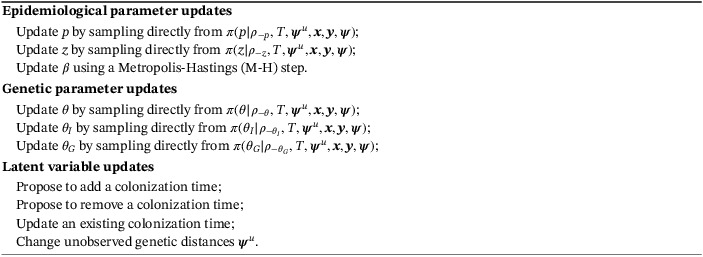

Updating the epidemiological and genetic parameters is fairly straightforward; these steps consist of Gibbs updates of all the corresponding parameters, except β, where a Gaussian random‐walk M‐H is employed instead. Updating *T* is much less straightforward. For example, proposing to add a colonization time is implemented by (i) selecting uniformly at random a currently uncolonized patient and propose that they become colonized, (ii) selecting a source of colonization from the set of colonized patients on this day, also uniformly at random, and (iii) drawing a set of proposed distances to every other sequence from every colonized patient. To update the genetic distances we first pick a patient uniformly at random from all those with one or more imputed sequences. We then pick one of their sequences, uniformly at random, and propose a new set of genetic distances according to the underlying genetic distance models.

We also perform additional updates, which we found improved the mixing of the Markov chain; in particular, updating the genetic parameters and distances simultaneously, swapping a patient and their source, and changing a source without changing colonization times.

Full details of all steps of the MCMC algorithm can be found in the supplementary material.

## 4 MODEL ASSESSMENT METHODS

Within the Bayesian framework, one natural way to undertake model assessment is to compare one or more summaries of the observed data with the corresponding quantities under the posterior predictive distribution. This is achieved by (i) fitting the model to data and generating samples from the posterior distribution of the model parameters ρ, (ii) simulating a number of new datasets using these samples as parameter values in the model, (iii) comparing the observed data summaries to the distribution of summaries obtained by simulation, typically checking whether or not the former lies within the central region or the tails of the latter.

For the epidemiological aspects of the data, suitable data summaries include the proportion of patients with a positive test result or with a positive test on admission.^1^ Although a similar approach can be taken for the genetic aspects of the data, we found that in practice this can be problematic. Specifically, we considered five summaries of the genetic data, namely, the mean, median, range, interquartile range, and sum of the genetic distances. In each case, we first simulated a number of datasets, then carried out the model fitting and assessment procedure, fitting both the true model used to create the dataset and also an alternative model with a different model for the genetic distances. We found that using these posterior predictive checks provided evidence against the fit of the wrong model, but also, for some datasets, gave evidence against the fit of the true model.^14^

A key reason why single summaries of genetic distance may be misleading is that the distances are conditional upon the transmission forest, and even simulating the correct model with the true parameter values may only rarely lead to a transmission forest compatible with the observed data. This motivates us to consider an alternative approach in which simulations are generated using samples from the posterior distribution of both ρ and the transmission forest described by *T*.

### 4.1 Model assessment for genetic distances

The following procedure produces *N* simulated sets of genetic distances ${\tilde{\psi }}_{1},\ldots ,{\tilde{\psi }}_{N}$ which can be compared with the observed data **ψ**. Suppose we have *M* posterior samples of (ρ,*T*) from the MCMC algorithm. We assume that the population of patients 𝒫 and the dates contained in ***y*** are the same as in the observed data.

1. For *k*=1,…,*N*, choose a posterior sample (ρ,*T*) uniformly at random from the *M* available.
2. Simulate a set of genetic distances **ψ**_*k*_ between all colonized patients using Θ, ***t*** ^*c*^, and ***s*** from (ρ,*T*).
3. Set ${\tilde{\psi }}_{k}$ as the restriction of **ψ**_*k*_ to the distances corresponding to those in **ψ**.

Note that step 3 is necessary because the transmission forest described by *T* may well include patients who do not correspond to any of the observed sequenced isolates. Conversely, since *T* has to be compatible with the observed data then for every ψ_*i*,*j*_∈**ψ**, there is a corresponding ${\tilde{\psi }}_{{\left(i,j\right)}_{k}}\in {\tilde{\psi }}_{k}$, *k*=1,…,*N*. Thus, each of ${\tilde{\psi }}_{1},\ldots ,{\tilde{\psi }}_{N}$ is a set of simulated distances for the same set of sequenced isolates as the data **ψ**.

In order to compare ${\tilde{\psi }}_{1},\ldots ,{\tilde{\psi }}_{N}$ with **ψ**, we assign each ψ_*i*,*j*_∈**ψ** a value α_*i*,*j*_ that describes how typical it is with respect to the distribution of simulated values ${\tilde{\psi }}_{i,j}=({\tilde{\psi }}_{{\left(i,j\right)}_{1}},\ldots ,{\tilde{\psi }}_{{\left(i,j\right)}_{N}})$. Ways to do this include a binary cutoff (eg, set α_*i*,*j*_ as the indicator function of the event that ψ_*i*,*j*_ lies within the 90% highest probability region of $\tilde{\psi }$) or setting α_*i*,*j*_ as the smallest α such that ψ_*i*,*j*_ lies within the (100×α)*%* highest probability region of $\tilde{\psi }$ (so the smaller α_*i*,*j*_, the more typical ψ_*i*,*j*_ is.) Finally, the set of α_*i*,*j*_ values can be presented graphically; an example is given below.

### 4.2 Simulation study

We conducted a brief simulation study to evaluate the model assessment method described above. Three datasets were simulated, with parameters as shown in Table 1. Admission dates for patients were chosen uniformly at random and independently from the study period, and each patient's length of stay was independently drawn from a Poisson distribution with a given mean. Swabs were taken from all patients on the ward every other day. Each positive swab was assumed to produce an observed sequence. Genetic distances were generated using either the Poisson error model or the Poisson chain model.

**Table 1:** Models and parameters used in simulation study

	Simulation 1	Simulation 2	Simulation 3
Study length (days)	100	200	100
Number of patients	100	200	100
Average length of stay (days)	7	5	7
True model	Poisson error	Poisson chain	Poisson error
*p*	0.06	0.06	0.06
*z*	0.8	0.8	0.8
β	0.01	0.02	0.02
θ	40	40	2
θ_*G*_	200	200	200
γ	30	—	40

**Figure 1:**
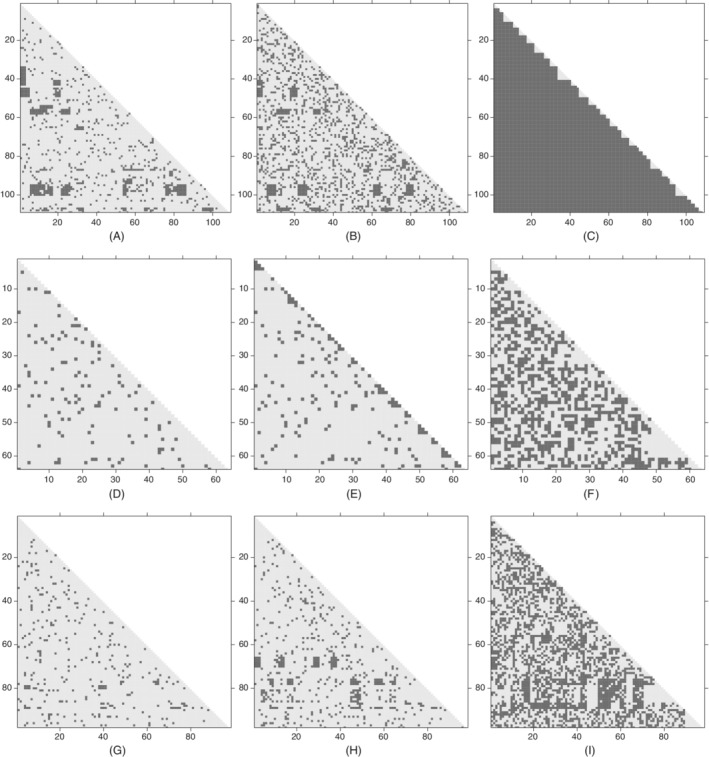
Results from simulation study on model assessment. The axes in each figure refer to the sequences, and each point shows whether the observed genetic distance between a sequence pair falls in the central 95% posterior predictive probability region (light shading) or not (dark shading). Each row shows results of fitting three models with true model (either Poisson error dependence or Poisson chain dependence) in first column, the other Poisson model in the second column and a geometric distribution version of the true model in the third column. Rows top to bottom correspond to simulations 1 to 3, respectively (see Table 1).

For each simulated dataset, we fitted three models, namely the two Poisson models and also a geometric model described in Section 5.2, and carried out the model assessment procedure for genetic distances defined in Section 4.1. The results are shown graphically in Figure 1, where we use a binary cutoff. Each subfigure shows, for each pair of sequences in the simulated data, whether or not the observed genetic distance lies within the central 95% posterior predictive probability region, with light shading used to indicate that it does. The first column shows results when the fitted model is the same as the model used to produce the simulated data, with the other columns showing results when the fitted model is different. It can be seen that the model assessment procedure is largely successful in identifying the true model in each case.

## 5 APPLICATION TO MRSA

### 5.1 Data

**Figure 2:**
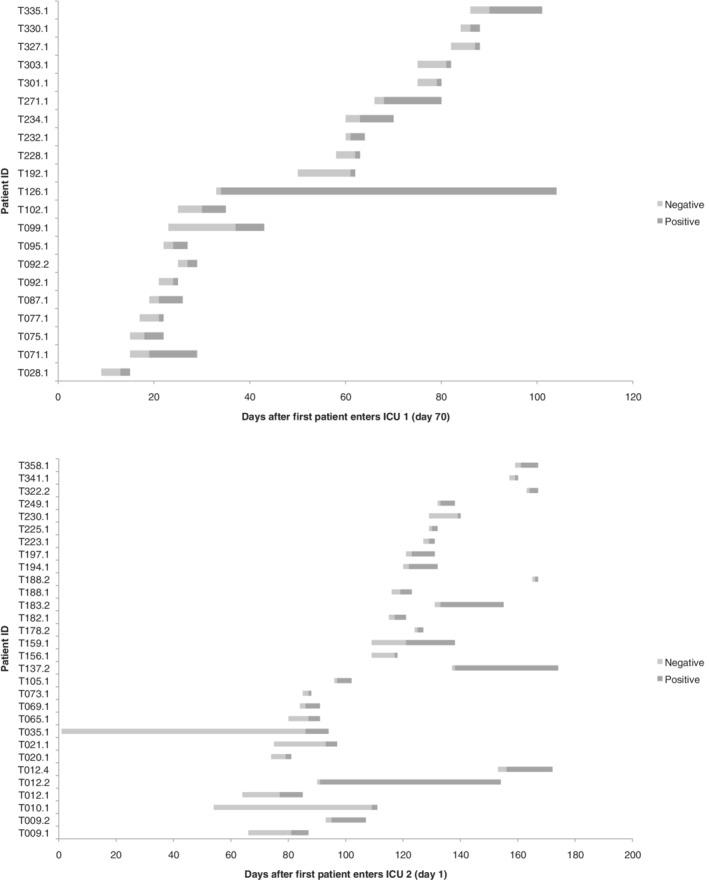
Methicillin‐resistant *Staphylococcus aureus* data: timelines for patients with a positive swab test. Each patient's line corresponds to their stay on the ward, with shading changing from light to dark on the date of their positive swab test. Day 1 and day 70 refer to real‐time days during the study.

We now apply our methods to data on an outbreak of MRSA in a hospital in Thailand in 2008. These data were collected during a study on two intensive care units (ICUs) in the same 1000 bed hospital in northeast Thailand.^15^ The data include 83 MRSA genome sequences from 51 distinct patients, which were aligned to a reference genome of the dominant lineage (ST 239 strain TW20) of MRSA in the hospital. A total of 2591 nucleotides changed from the reference genome in at least one patient sequence. The data were collected by repeat screening for MRSA of patients on two ICUs, one surgical and one pediatric, over 3 months. Table 2 summarizes the data from each ICU and Figure 2 displays timelines for each of the patients who ever had a positive swab test. Further details of the dataset can be found in Tong et al.^15^

**Table 2:** Summary of the MRSA data

	ICU 1	ICU 2
Ward type	Surgery	Pediatric
Number of patients admitted	170	114
Number of distinct patients	169	98
Number of patients with at least one positive swab	20	29
Total number of positive swabs collected	51	89
Total number of swabs sequenced	43	40

Abbreviations: ICU, intensive care unit; MRSA, methicillin‐resistant *Staphylococcus aureus*.

### 5.2 Models

We initially fitted the two Poisson distribution models for genetic distance defined in Sections 2.4.1 and 2.4.2. As shown below, these models did not provide a convincing fit to the data and so we also developed four additional models. These four models have the same assumptions as the Poisson models for distances between sequences in a chain of transmission with transmission distance *k*(*i*,*j*)>1, but differ by having alternative distributions for other distances. In particular we used geometric distributions, as employed in Worby et al,^1^ and negative binomial distributions, to allow separate modelling of the mean and variance of those genetic distances that were not well described by one‐parameter distributions. For each distribution, we considered both error dependence and chain dependence versions. A summary of all six models is given in Table 3.

**Table 3:** Distribution of the genetic distance Ψ_*i*,*j*_ between sequences *i* and *j* for the six models used for the methicillin‐resistant *Staphylococcus aureus* data analysis

	Poisson models	Geometric models	Negative binomial models
*k*(*i*,*j*)=∞	Pois(θ_*G*_)	Geom(*φ*_*G*_)	NB($\mu_{G},\sigma_{G}^{2}$)
*k*(*i*,*j*)=0	Pois(θ_*I*_)	Geom(*φ*_*I*_)	Geom(*φ*_*I*_)
*k*(*i*,*j*)=1	Pois(θ)	Geom(*φ*)	NB(μ,σ^2^)

*Note: k*(*i*,*j*) is the transmission distance between *i* and *j* as defined in Section 2.3, Pois(θ) is a Poisson distribution with mean θ, Geom(*φ*) is a geometric distribution with mean *φ* ^−1^ and NB(μ,σ^2^) is a negative binomial distribution with mean μ and variance σ^2^. For *k*(*i*,*j*)>1, all models use the distributions specified in Equations (1) or (2).

We assigned uninformative prior distributions to the model parameters; full details are given in the supplementary material.

### 5.3 Results

Table 4 contains results from all six models for the epidemiological parameters. There is reasonable agreement across all models, particularly for the transmission parameter β and test sensitivity *z*, the latter being around 70% for ward 1 and 80% for ward 2. The proportion of patients estimated to be colonized on admission shows more variability between models, ranging from 3% to 6% on ward 1 and from 3% to 12% on ward 2.

**Table 4:** Methicillin‐resistant *Staphylococcus aureus* data: posterior means and equal‐tailed 95% credible intervals for the epidemiological parameters

	Ward 1			Ward 2		
Model	*p*	*z*	β	*p*	*z*	β
Poisson error	0.048	0.72	0.013	0.067	0.79	0.010
	(0.02,0.09)	(0.59,0.83)	(0.007,0.021)	(0.028,0.12)	(0.68,0.84)	(0.006,0.014)
Poisson chain	0.049	0.71	0.012	0.033	0.81	0.013
	(0.019,0.092)	(0.58,0.81)	(0.007,0.019)	(0.007,0.076)	(0.71,0.90)	(0.008,0.019)
Geometric error	0.060	0.68	0.015	0.10	0.78	0.011
	(0.024,0.11)	(0.56,0.80)	(0.008,0.025)	(0.04,0.19)	(0.68,0.88)	(0.006,0.016)
Geometric chain	0.034	0.69	0.017	0.11	0.84	0.011
	(0.01,0.071)	(0.57,0.80)	(0.009,0.027)	(0.053,0.19)	(0.74,0.91)	(0.007,0.018)
Neg bin error	0.038	0.72	0.016	0.084	0.83	0.012
	(0.013,0.076)	(0.60,0.83)	(0.009,0.024)	(0.036,0.15)	(0.74,0.90)	(0.007,0.018)
Neg bin chain	0.030	0.71	0.016	0.12	0.79	0.011
	(0.008,0.066)	(0.59,0.82)	(0.009,0.024)	(0.057,0.20)	(0.70,0.87)	(0.006,0.017)

It is of interest to see how much the whole‐genome‐sequence data tell us about the epidemiological parameters. It is possible to fit the underlying transmission model without using any genetic data, and this yields posterior mean estimates (*p*,*z*,β)=(0.046,0.759,0.012) and (0.193,0.862,0.007) for wards 1 and 2, respectively.^16^ The sensitivity and transmission rate parameters are broadly similar to those in Table 4, but in ward 2, the probability of being colonized on admission is far higher if the genetic data are ignored. In this case, the genetic data thus suggest more within‐ward transmission than that inferred from epidemiological data alone. The probability of being colonized on admission and the within‐ward transmission rate are typically negatively correlated a posteriori when estimated solely by epidemiological data, since they represent competing ways of explaining the test results, and our results show that the whole‐genome‐sequence data provide a way of partially resolving this issue.

Table 5 shows genetic parameter estimates for all six models. The estimates for mean genetic distance for within‐patient isolates in a given ward are comparable across all models. The corresponding variances are determined by the mean values, since the underlying assumed distribution is either Poisson or geometric. The mean estimates for distances between patients in a given ward who are in different transmission trees are broadly comparable. Again, the corresponding variances are determined for the Poisson and geometric models, but for the negative binomial models, the variance can be estimated separately and found to be considerably different from the Poisson models. This suggests that the Poisson models fit the data less well in this respect. Similar conclusions hold for the parameters associated with direct transmission, although here the mean values are less similar across the three model types.

**Table 5:** Methicillin‐resistant *Staphylococcus aureus* data: posterior means and equal‐tailed 95% credible intervals for the mean and variance of the genetic distance between sequenced isolates that are in different transmission chains (separate), taken from the same patient (within‐patient) or taken from patients directly connected in a transmission tree (direct)

	Separate		Within‐patient		Direct	
Model	Mean	Variance	Mean	Variance	Mean	Variance
*Ward 1*						
Poisson error	380.9	380.9	37.2	37.2	39.6	39.6
	(378.8,383.2)	(378.8,383.2)	(36.3,38.1)	(36.3,38.1)	(38.1,41.1)	(38.1,41.1)
Poisson chain	380.6	380.6	37.2	37.2	40.2	40.2
	(378.9,382.2)	(378.9,382.2)	(36.3,38.1)	(36.3,38.1)	(39.1,41.4)	(39.1,41.4)
Geometric error	367.1	1.35 ×10^5^	38.2	1.43 ×10^3^	47.5	2.30 ×10^3^
	(335.1,401.5)	(1.12,1.61)×10^5^	(33.1,44.2)	(1.06,1.91)×10^3^	(32.5,69.0)	(1.02,4.70)×10^3^
Geometric chain	369.2	1.36 ×10^5^	38.2	1.43 ×10^3^	93.7	9.63 ×10^3^
	(338.7,402.2)	(1.15,1.62)×10^5^	(33.0,44.1)	(1.06,1.92) ×10^3^	(48.3,134.6)	(2.52,18.1)×10^3^
Neg bin error	386.1	4.72 ×10^4^	38.2	1.43 ×10^3^	120.5	1.95×10^4^
	(365.6,406.8)	(3.52,5.82)×10^4^	(33.1,44.3)	(1.10,1.92)×10^3^	(96.7,155.5)	(1.15,3.50)×10^4^
Neg bin chain	383.60	4.55 ×10^4^	38.2	1.43×10^3^	131.5	2.38×10^4^
	(364.1,404.0)	(3.82,5.40)×10^4^	(33.0,44.2)	(1.10,1.92)×10^3^	(106.7,157.1)	(1.41,3.63)×10^4^
*Ward 2*						
Poisson error	339.0	339.0	8.0	8.0	52.3	52.3
	(337.1,341.2)	(337.1,341.2)	(6.33,9.84)	(6.33,9.84)	(50.3,54.3)	(50.3,54.3)
Poisson chain	212.0	212.0	8.0	8.0	61.7	61.7
	(209.5,214.5)	(209.5,214.5)	(6.32,9.87)	(6.32,9.87)	(59.3,68.4)	(59.3,68.4)
Geometric error	223.6	4.99 ×10^4^	9.11	80.6	65.6	2.34 ×10^3^
	(204.7,243.1)	(4.17,5.88) ×10^4^	(5.18,16.1)	(21.6, 231.7)	(41.2,124.6)	(1.09,4.69) ×10^3^
Geometric chain	222.0	4.92 ×10^4^	9.10	81.9	42.0	2.02 ×10^3^
	(204.7,240.0)	(4.18,5.73)×10^4^	(5.16,16.1)	(21.6,242.9)	(20.5,79.5)	(4.00,62.7)×10^3^
Neg bin error	217.7	6.70 ×10^4^	9.10	82.2	55.2	2.51 ×10^3^
	(196.7,240.3)	(5.29,8.35) ×10^4^	(5.18,16.3)	(21.7,248.5)	(41.6,76.2)	(1.10,6.17) ×10^3^
Neg bin chain	208.0	5.81 ×10^4^	9.09	81.8	183.2	9.27 ×10^4^
	(190.0,228.9)	(4.72,7.22) ×10^4^	(5.18,16.1)	(21.8,237.6)	(95.4,365.2)	(1.81,37.2) ×10^4^

**Figure 3:**
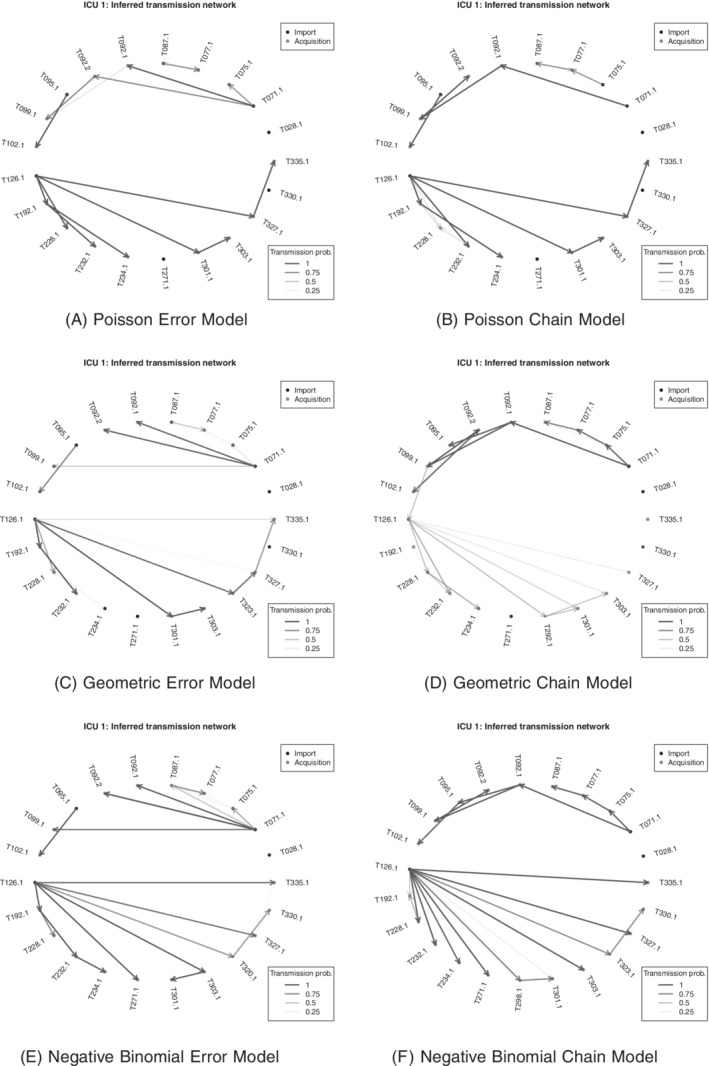
Methicillin‐resistant *Staphylococcus aureus* data: estimated transmission forest under each model for ward 1

**Figure 4:**
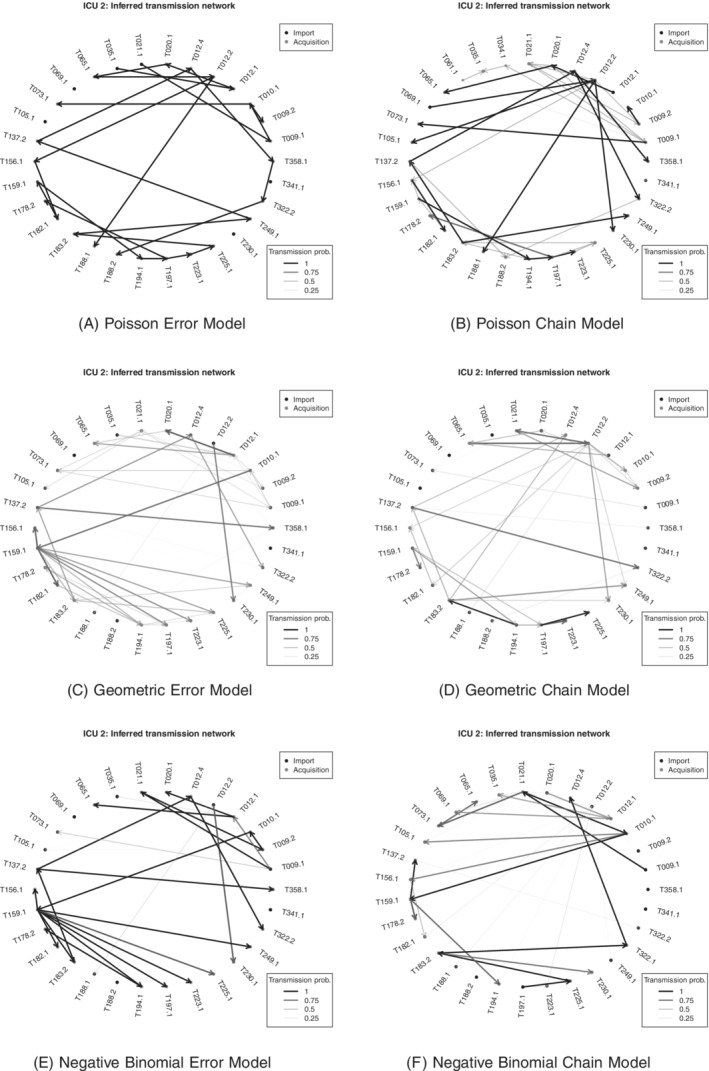
Methicillin‐resistant *Staphylococcus aureus* data: estimated transmission forest under each model for ward 2

Figures 3 and 4 show inferred transmission forests for each model. Broadly speaking, the error dependence and chain dependence versions of each model give similar results, whereas more variation is seen across the three different distributions. In particular, the negative binomial models suggest slightly more transmission within the ward, and fewer imported cases, than the Poisson or geometric models. This is most likely due to the fact that the former allow for greater variance in the genetic distances in direct transmission, which in turn makes such transmission more likely given the observed data.

Within ward 1, all models suggest that there are two principle transmission chains, initiated by patients T0771.1 and T126.1. Patient T126.1 in particular appears to be the source of colonization for numerous other patients; one possible reason for this is that particular patient was present on the ward for far longer than any of the others. A previous analysis, using completely different methods, also found patient T126.1 to be responsible for many of the colonization events.^15^ Within ward 2, the results are more variable across models, although there is still evidence of patients who act as the source of colonization for several other patients, such as patients T012.2 and T159.1, the former again being present on the ward for longer than most other patients.

### 5.4 Model assessment

We carried out model assessment of both the epidemiological and genetic aspects of the models. For the former, we first compared the observed total number of patients with a positive swab test result, namely, 30 patients in ward 1 and 22 in ward 2, with the corresponding number obtained from the posterior predictive distribution. Specifically, we performed 1000 simulations of each model with all admission, discharge and test dates fixed to the known values from the data, with parameters drawn from the posterior distribution, that is, from the MCMC algorithm output for the model in question. Table 6 shows 95% probability intervals from the simulations, all of which contain the observed values.

**Table 6:** Methicillin‐resistant *Staphylococcus aureus* data: 95% highest posterior predictive probability regions for the total number of patients to have a positive swab. The observed values were 30 patients for ward 1 and 22 patients for ward 2

Model	Ward 1	Ward 2
Poisson error	(7,53)	(5,28)
Poisson chain	(2,33)	(1,32)
Geometric error	(2,53)	(4,39)
Geometric chain	(1,34)	(7,49)
Neg bin error	(7,56)	(6,33)
Neg bin chain	(1,31)	(7,44)

**Figure 5:**
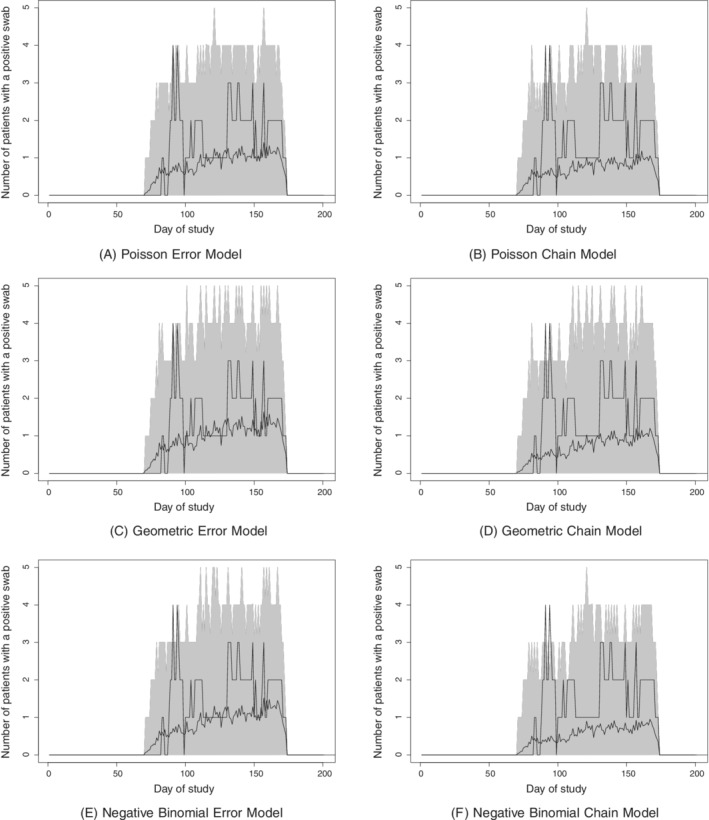
Posterior prediction of the number of patients on the ward with a positive swab over time under each model for ward 1. The black step‐function‐like line shows the observed data, the rapidly‐varying line shows the posterior predictive mean, and the shaded area is the posterior predictive 95% probability interval.

**Figure 6:**
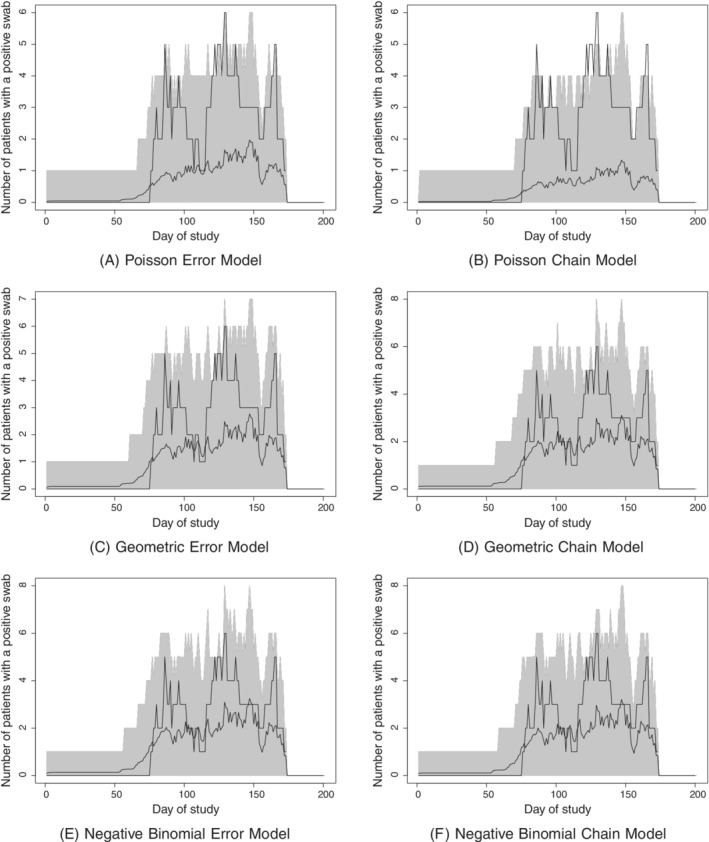
Posterior prediction of the number of patients on the ward with a positive swab over time under each model for ward 2. The black step‐function‐like line shows the observed data, the rapidly‐varying line shows the posterior predictive mean, and the shaded area is the posterior predictive 95% probability interval.

We next considered a time‐dependent quantity for model assessment, namely, the number of patients on the ward on a given day who have had a positive swab on that day or any previous day. Figures 5 and 6 show 95% probability intervals from the simulations. In each case, the observed data lie well within the probability intervals for all, or all but a few days, and so there is no material evidence against any of the models.

**Figure 7:**
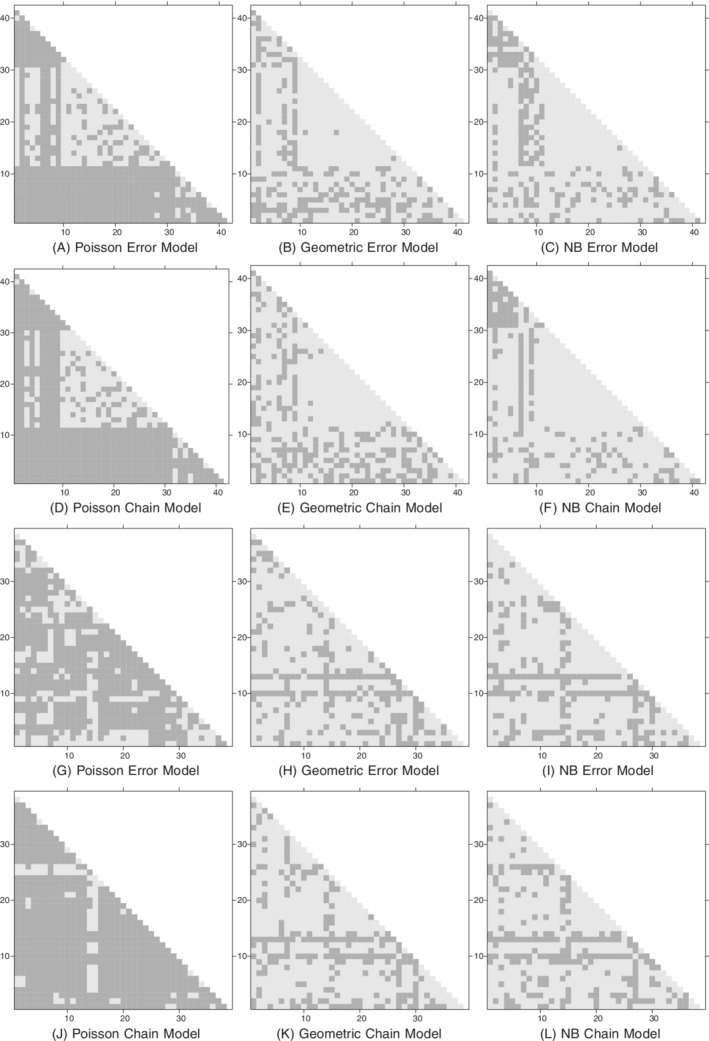
Methicillin‐resistant *Staphylococcus aureus* data: model assessment using methods described in main text. The axes in each figure refer to the observed sequences, and each point shows whether the observed genetic distance between a sequence pair falls in the central 95% posterior predictive probability region (light shading) or not (dark shading). A‐F, Ward 1. G‐L, Ward 2

To assess the genetic part of the model, we used the method described in Section 4. Figure 7 shows results based on 1000 genetic distance matrices drawn from the posterior predictive distribution for each model. It is clear that the Poisson models have inferior model fit compared with the geometric and negative binomial models, with the latter providing a reasonable fit to the data.

## 6 CONCLUSION AND DISCUSSION

We have developed new models for analyzing whole‐genome‐sequence data by introducing natural dependencies into the class of models developed by Worby et al.^1^ In addition we have developed model assessment methods that provide a means for quantifying how well the models fit the genetic data. Although we have focused on nosocomial pathogens, the methods themselves are generic in nature and could easily be adapted to other infectious disease settings.

Whole‐genome‐sequence data offer the potential to reconstruct transmission pathways in a disease outbreak with less uncertainty than that provided by standard epidemiological data alone. In healthcare settings, one clinically important consequence is that it becomes more feasible to accurately identify which cases have arisen due to internal transmission as opposed to being imported cases. Such information can be used to inform infection control policies and procedures.

We used Poisson, geometric, and negative binomial distributions for genetic distance models. Choosing which distributions to use can be dependent on the dataset under consideration, although in our experience there is often little material difference in the resulting inference for who‐infected‐whom. There is some loose justification for the use of Poisson distributions insofar as the genetic mutations counted by SNPs could be reasonably thought of as rare events, for which the Poisson distribution is a standard modelling choice. However, SNP data themselves arise via complex sequencing procedures, and hence the distributions in our models are effectively attempting to capture the output from the combination of underlying biological mechanisms and laboratory methods.

The genetic distance models employed in this article do not make explicit use of time, but instead depend on the number of links along transmission chains. However, it is natural to suppose that the genetic distances along a transmission chain may depend on the times between successive colonization events. We found that incorporating this idea into our models had little material impact on the results for the MRSA data.^14^ One reason for this is that most patients only remain in the ward for a few days, so there is relatively little variability in the times between successive colonization events, and thus the number of links in the transmission chain is almost as informative as the times themselves.

Our models are defined in discrete‐time, although our methods can equally be applied to continuous‐time models.^14^ For hospital infection models, small estimation biases can arise if a discrete‐time model is used in a setting where the data are assumed to be generated from a continuous‐time model,^17^ although some of the underlying assumptions in the transmission mechanisms of both discrete‐time and continuous‐time models are questionable in reality. For instance, continuous‐time models typically assume that transmission potentially occurs at any time of day or night, but most ICUs see more potential colonization opportunities during the day as healthcare workers, other staff, and visitors are far less likely to be active on the ward during the night. Conversely, discrete‐time models aggregate events together into time units such as days, but this simplification can be unrealistic, particularly if multiple colonization events are likely to occur within one time unit. For the MRSA data we have considered, there are relatively a few colonization events, which helps motivate our choice of discrete‐time models.

We have assumed that if individuals become colonized then they remain so for the duration of their time on the hospital ward. This is a fairly common assumption^13^, ^18^, ^19^ and is reasonable for wards such as ICUs where patient stays are typically fairly short, and in particular likely to be shorter than the time taken for clearance of pathogen carriage. However, the methods we have described could equally be applied to models that include carriage clearance, and also readmission of patients, since the data‐augmentation methods keep track of the required information such as the transmission forest.

## DATA ACCESSIBILITY

The MRSA data used in this article are not freely available. Please contact the corresponding author for more information.

## Supporting information

Data S1: Supporting Information.
