# Supplementary material for: Modelling, Bayesian inference, and model assessment for nosocomial pathogens using whole‐genome‐sequence data
Source: Stat Med. 2020 Mar 6;39(12):1746–65. doi: 10.1002/sim.8510 (PMC7217057; doi:10.1002/sim.8510)
Supplement: Supplementary file 1 — Data S1: Supporting Information. [file SIM-39-1746-s001.pdf]

Modelling, Bayesian inference and model assessment for nosocomial pathogens using whole-genome-sequence data
Supplementary Material

R. Cassidy*, T. Kypraios*, P. D. O'Neill*

1 Markov chain Monte Carlo algorithm

Here we describe an MCMC algorithm to sample from the posterior density

$$\pi(\rho, T, \boldsymbol{\psi}^u | \boldsymbol{x}, \boldsymbol{y}, \boldsymbol{\psi}) \propto \pi(\boldsymbol{x}, \boldsymbol{\psi}, \boldsymbol{\psi}^u | \rho, T, \boldsymbol{y}) \pi(T | \rho, \boldsymbol{y}) \pi(\rho),$$

where $\rho = \{p, \beta, z, \Theta\}$, assuming the Poisson versions of the models. The algorithms for the Geometric and Negative Binomial versions are similar. The MCMC algorithm consists of updates for each parameter, which are themselves sufficient to define a Markov chain with the required stationary distribution, plus some additional updates which we found improved the mixing of the Markov chain. All assigned prior distributions are assumed to be mutually independent. We use the notation a_{-b} to denote a quantity a with component b removed, e.g. $\rho_{-p} = \{\beta, z, \Theta\}$.

1.1 Epidemiological parameter updates

The importation probability p can be updated according to its full conditional distribution. We assume that $p \sim Beta(\alpha_p, \beta_p)$ *a priori* where $Beta(a, b)$ denotes a Beta distribution with probability density function $f(x) \propto x^{a-1}(1-x)^{b-1}$, $0 \leq x \leq 1$. It follows from (5) that

$$p | \rho_{-p}, T, \boldsymbol{\psi}^u, \boldsymbol{x}, \boldsymbol{y}, \boldsymbol{\psi} \sim Beta\left(\alpha_p + \sum_i \phi_i, \beta_p + \sum_i (1 - \phi_i)\right).$$

In the MRSA analysis we set $\alpha_p = \beta_p = 1$.

Similarly, if $z \sim Beta(\alpha_z, \beta_z)$ *a priori* then, from (4)

$$z | \rho_{-z}, T, \boldsymbol{\psi}^u, \boldsymbol{x}, \boldsymbol{y}, \boldsymbol{\psi} \sim Beta\left(\alpha_z + \text{TP}, \beta_z + \text{FN}\right).$$

For the MRSA analysis we set $\alpha_z = \beta_z = 1$.

The infection parameter β is assumed to have an improper prior distribution on $(0, \infty)$, and does not have a standard full conditional distribution and so it is updated using a Gaussian random walk Metropolis-Hastings step.

*School of Mathematical Sciences, University of Nottingham

1.2 Genetic parameter updates

We assume that the direct-transmission parameter θ has a $\Gamma(\nu, \lambda)$ prior distribution, i.e. with probability density function $f(x) \propto x^{\nu-1} \exp(-\lambda x)$ for $x > 0$. It follows from (2) and (1) that for both the error dependence and chain dependence model we have

$$\theta|\rho_{-\theta}, T, \boldsymbol{\psi}^u, \boldsymbol{x}, \boldsymbol{y}, \boldsymbol{\psi} \sim \Gamma\left(\sum_{(i,j)\in\mathcal{K}_1} \psi_{ij} + \nu, |\mathcal{K}_1| + \lambda\right),$$

where $\mathcal{K}_1 = \{(i,j) \in \mathcal{S} : k(i,j) = 1\}$ is the set of pairs of sequences with transmission distance 1, and thus θ can be updated directly from its full conditional distribution. In a similar fashion, if $\theta_G \sim \Gamma(\nu_G, \lambda_G)$ and $\theta_I \sim \Gamma(\nu_I, \lambda_I)$, *a priori* and $\mathcal{K}_\infty$ and $\mathcal{K}_0$ are defined in the obvious manner, we obtain

$$\theta_G|\rho_{-\theta_G}, T, \boldsymbol{\psi}^u, \boldsymbol{x}, \boldsymbol{y}, \boldsymbol{\psi} \sim \Gamma\left(\sum_{(i,j)\in\mathcal{K}_\infty} \psi_{ij} + \nu_G, |\mathcal{K}_\infty| + \lambda_G\right),$$

and

$$\theta_I|\rho_{-\theta_I}, T, \boldsymbol{\psi}^u, \boldsymbol{x}, \boldsymbol{y}, \boldsymbol{\psi} \sim \Gamma\left(\sum_{(i,j)\in\mathcal{K}_0} \psi_{ij} + \nu_I, |\mathcal{K}_0| + \lambda_I\right).$$

For the MRSA analysis, all these three genetic parameters were assigned independent $\Gamma(1, 10^{-6})$ prior distributions.

Finally, the parameter γ in the error dependence models is assumed to have an improper prior distribution on $(0, \infty)$, and does not have a standard full conditional distribution and so it is updated using a Gaussian random walk Metropolis-Hastings step.

1.3 Latent variable updates

Recall that the latent (i.e. unobserved) variables are the colonisation times $\boldsymbol{t}^c$, the colonised-on-admission indicator functions $\boldsymbol{\phi}$, the sources of colonisation $\boldsymbol{s}$ and the unobserved genetic distances $\boldsymbol{\psi}^u$. We now describe four update steps which together will update these quantities, where one update step is chosen with equal probability during each iteration of the MCMC algorithm. We define the following quantities.

- n_{sus} is the number of patients that never have a positive test;
- n_{add} is the number of patients that never have a positive test and that are colonised;
- n_{add0} is the number of patients that never have a positive test, are colonised and are not the source of colonisation for any other patient.

For each update step below we give the Metropolis-Hastings proposal ratio q_{T,T^*}; the acceptance probability for each step is then

$$\min\left(1, \frac{\pi(\boldsymbol{x}, \boldsymbol{\psi}, \boldsymbol{\psi}^u|\rho, T^*, \boldsymbol{y})\pi(T^*|\rho, \boldsymbol{y})}{\pi(\boldsymbol{x}, \boldsymbol{\psi}, \boldsymbol{\psi}^u|\rho, T, \boldsymbol{y})\pi(T|\rho, \boldsymbol{y})} q_{T,T^*}\right).$$

Add a colonisation event In this move we select uniformly at random a currently uncolonised patient, i, and propose that they become colonised. If there are no uncolonised patients to choose

from then no move is made. The number of uncolonised patients to choose from equals $n_{sus} - n_{add}$. With probability w the chosen patient is proposed to be colonised before admission to the ward, so they are an importation. With probability $1 - w$ the patient is proposed to be colonised by another colonised patient on the ward. In this case we draw a day of colonisation, t_i^{c*} from $\left\{t_i^a, t_i^a + 1, \ldots, t_i^d\right\}$. We select a source of colonisation uniformly at random from the set of colonised patients on this day. If there are no available patients to be a source on this day, the move is not made. If the move is possible, whether we are inferring an importation or a colonisation on the ward, we then draw a set of proposed genetic distances, $\left\{\psi^*_{\sigma(i),j}\right\}$, from patient i's sequence, $\sigma(i)$, to every other sequence from every colonised patient (both those observed positive in the data, either with or without sequenced isolates, and those currently added by the algorithm). The distances are drawn from the relevant probability distributions depending on the choice of genetic distance model, and the transmission distances $k(\sigma(i), j)$.

Using the 'remove colonisation' step definition below, the proposal ratio for adding an importation is

$$q_{T,T^*} = \frac{n_{sus} - n_{add}}{w(1 + n_{add0})A^*_{\sigma(i)}(\Theta)},$$

where

$$A^*_{\sigma(i)}(\Theta) = \prod_j P[\Psi_{\sigma(i),j} = \psi^*_{\sigma(i),j}|\Theta]$$

is the probability of proposing the genetic distances associated with the proposed new importation. The proposal ratio for adding an acquisition (i.e. a patient colonised on the ward) is

$$q_{T,T^*} = \frac{(n_{sus} - n_{add})(t_i^d - t_i^a + 1)C(t_i^{c*})}{(1 - w)(1 + n_{add0})A^*_i(\Theta)}.$$

Remove a colonisation event In this step we select uniformly at random one of the n_{add0} currently added colonised patients who are not the source of any other colonisations and propose that they be no longer colonised. This move cannot be made if no such individuals exist. If we propose to remove the colonisation time from patient i who is an importation then the proposal ratio is

$$q_{T,T^*} = \frac{w n_{add0} A_{\sigma(i)}(\Theta)}{n_{sus} - n_{add} + 1},$$

where

$$A_{\sigma(i)}(\Theta) = \prod_j P[\Psi_{\sigma(i),j} = \psi_{\sigma(i),j}|\Theta]$$

is the probability of the genetic distances associated with patient i. The proposal ratio when we propose to remove the colonisation time for a patient i who was colonised on the ward is

$$q_{T,T^*} = \frac{(1 - w)n_{add0}A_{\sigma(i)}(\Theta)}{(n_{sus} - n_{add} + 1)(t_i^d - t_i^a + 1)(C(t_i^c) - 1)}.$$

Move a colonisation time In this move we pick a patient i uniformly at random from the set of currently colonised patients and move their colonisation time. As when we added a colonisation time, we propose that the patient was positive on admission with probability w. With probability $1 - w$ the patient acquired the pathogen whilst on the ward so we sample a colonisation time uniformly at random from the set $\{t_i^a, t_i^a + 1, \ldots, f_i\}$, where f_i is the latest day on which patient i could have been colonised. This is the minimum of (i) the day before the patient's first positive

swab result, if any; (ii) the day before the colonisation of the first patient to be colonised on the ward by i, if any; (iii) the day of discharge t_i^d. We then propose a source uniformly at random for patient i from the set of colonised patients on the chosen day of colonisation. If there are no such colonised patients no move is made.

If patient i is colonised on the ward on day t_i^c and we propose that they be colonised on the ward on day t_i^{c*} then we have

$$q_{T,T^*} = \frac{C(t_i^{c*})}{C(t_i^c)}.$$

If patient i is colonised on the ward on day t_i^c and we propose that they be colonised on admission then we have

$$q_{T,T^*} = \frac{1-w}{w(f_i - t_i^a + 1)C(t_i^c)}.$$

If patient i is colonised on admission and we propose that they be colonised on the ward on day t_i^{c*} then

$$q_{T,T^*} = \frac{w(f_i - t_i^a + 1)C(t_i^{c*})}{1-w}.$$

Finally, if patient i is colonised on admission and we propose that they remain colonised on admission then nothing has changed and the update step is complete.

Change genetic distances In this move we attempt to update genetic distances other than those found in the data set, i.e. distances which are imputed in the algorithm. To do so we pick a patient i uniformly at random from all those with one or more imputed sequences, and then pick one of their sequences, $L(i)$ say, uniformly at random. We then propose a new set of genetic distances $\left\{\psi^{u*}_{L(i),j}\right\}$ according to the underlying genetic distance model. The proposal ratio is

$$q_{\boldsymbol{\psi}^u,\boldsymbol{\psi}^{u*}} = \frac{A_{L(i)}(\Theta)}{A^*_{L(i)}(\Theta)}.$$

1.4 Additional updates to improve mixing

Block updates for genetic parameters and distances All the genetic parameter updates described above are dependent on the current values of the genetic distances $\psi_{i,j}$, and in particular those which have been imputed rather than being part of the data. This correlation can cause mixing problems and we found it was beneficial for some models to update θ and θ_G with some genetic distances, as follows. First, proposed new values θ^* and θ_G^* are obtained using Gaussian proposals centered on the current values. Second, we pick a patient i and a sequence $L(i)$ in the manner described in the 'change genetic distances' move above. Third, we propose new genetic distances by using the underlying genetic distance model, but using the proposed parameters θ^* and θ_G^*. The proposal ratio is

$$q_{\boldsymbol{\psi}^u,\boldsymbol{\psi}^{u*}} = \frac{A_{L(i)}(\Theta)}{A^*_{L(i)}(\Theta^*)}.$$

Single updates for genetic distances Rather than proposing to update all of the genetic distances associated with patient i, which can lead to low acceptance probabilities if the move is too large, an alternative is to pick one of i's distances uniformly at random and then, with equal probability, add or subtract a quantity drawn from a pre-specified distribution, such as a Poisson distribution. The proposal ratio for this move equals 1.

Swap a patient and their source The motivation behind this move is to provide a small change to the overall transmission forest, since large-scale moves can have low acceptance probabilities. A patient j is selected uniformly at random from those who are colonised on the ward. Their source, patient i, is identified. If i colonises another patient before j then no move is made. If $t_i^c < t_j^a$ no move is made. Otherwise we propose $t_i^{c*} = t_j^c$, $t_j^{c*} = t_i^c$, and $s_i^* = j$. The proposal ratio for this move equals 1.

Change a source without changing colonisation time Another small move is as follows. We select a patient i uniformly at random from those colonised on the ward. We uniformly at random select a patient j from those colonised on day t_i^c, and propose $s_i^* = j$. The proposal ratio for this move equals 1.
